# Gender and health inequalities: intersections with other relevant axes of oppression

**DOI:** 10.3402/gha.v8.30292

**Published:** 2015-11-30

**Authors:** Carmen Vives-Cases, Malin Eriksson, Isabel Goicolea, Ann Öhman

**Affiliations:** 1 Department of Community Nursing, Preventive Medicine and Public Health and History of Science, Alicante University, Alicante, Spain; 2 CIBER of Epidemiology and Public Health, Barcelona, Spain; 3 Epidemiology and Global Health Unit, Department of Public Health and Clinical Medicine, Faculty of Medicine, Umeå University, Umeå, Sweden; 4 Umeå Centre for Gender Studies, Umeå University, Umeå, Sweden

The risk of disease, disability, and mortality as well as access to health services are unfairly distributed among the population, with certain groups bearing an unequally larger burden of ill health and poorer access to care due to gender, sexual identity/orientation, ethnic background, or class. According to the WHO Commission on Social Determinants of Health (CSDH), these health inequalities emanate from socioeconomic and political factors (governance, cultural values, macroeconomic policies), which generate a set of socioeconomic positions in society according to which populations are stratified based on gender, ethnicity, education, income, or other factors. These societal inequalities influence people's material and psychosocial circumstances as well as behavioral and biological factors, which in turn impact on health inequalities (1). Tackling gender, race/ethnic, and socioeconomic inequalities in society is thus recognized as the most powerful action to cope with unequal health risks distribution, and social innovations focusing on these ‘root causes’ are needed in order to prevent and stop endemic social inequalities and social exclusion in health within low-income as well as high-income countries (2). Increasing existing knowledge and making visible the health status of the most vulnerable and invisible groups are critical in order to contribute to this imperative challenge.

Gendered power relations have been identified among the most influential social determinants of health inequalities due to their damaging effects on women's and men's health at different levels over their lifetime (3). Last year, *Global Health Action* presented for the first time a call for articles on Gender and Health aimed to include a variety of empirical and theoretical perspectives, among them sexual and reproductive health and rights, gender-based violence, ageing and gender, health systems, climate change, and globalization; all with respect to gender. A total of 19 articles were published and the closing editorial for that special issue, entitled ‘Gender and health – aspects of importance for understanding health and illness in the world’, pointed out the most prevalent topics and also hinted at gaps and lacking perspectives (4). One of the identified gaps was the lack of available studies tackling the complex interaction between gender and other markers of inequalities in understanding and targeting health inequalities. The complex nature of these interactions usually limits our knowledge about gender and social inequalities in health.

As has been introduced in different theoretical frameworks, notably feminist theory and social epidemiology, gender inequalities in health in the field of violence, cardiovascular disease, or HIV/AIDS risk among other issues cannot be reduced to being a matter of gendered power relations. They are inseparably amplified by other axes of social stratification and oppression related to racism, classism, heterosexism, or ageism (5). Three (or more) dimensional lenses are needed to develop a more comprehensive research approach to these social inequalities in health. The complex interaction between gender (in)equality and health (inequality) also deserves further research. On the one hand, gender inequality has been strongly connected with harmful health effects for men, children, and women (3). On the other hand, the deteriorating mental health of young women in countries that rank highest for gender equality calls for further research on the connections between gender (equality), health, age, and other conditions as well as the gaps between policy achievements and lived experiences (6, 7). The question of whether increased gender equality will (always) come hand-in-hand with better health for all deserves still further exploration.

The current call for papers entitled ‘Gender and health inequalities: intersections with other relevant axes of oppression’ aims to generate knowledge about how gender inequalities in health/disease/mortality/and access to health care systems interact with other important axes of oppression (race/ethnicity, social class, religion, and/or migratory status, among others) through different levels of power (from the global to the local) at different lifetime stages for a population. It also aims to contribute to a better understanding of the relationship between gender (in)equalities and health (inequalities). We welcome different types of contributions: empirical research, theoretical papers, methodological papers, and reviews. Studies aiming to contribute to developing gender and social theories building on intersectional, ecosocial, relational, or biosocial approaches are welcome. Also of interest are methodological papers using qualitative, quantitative, or mixed methods, and are particularly studies that explore means of better addressing the complexity of analyzing health inequalities according to this multidimensional or *multiple approach* (8).

Papers about the effects of gender, ethnicity, ageing, migration status, and other relevant social determinants in the distribution of prevalent, emergent, or neglected disease are of interest in the context of this new special issue as well as those that analyze the interaction of different axes of oppression with other intermediate social determinants related to work conditions, access to health care systems, climate change, social capital, or other determinants. We also welcome papers that address not only issues of dominance and/or suffering but also those about resistance, agency, resilience, and/or empowerment. We encourage submissions from researchers working in low-, middle-, and high-income countries.

The guest editors for this call ‘Gender and health inequalities: intersections with other relevant axes of oppression’ are Carmen Vives-Cases and Ann Öhman. Carmen Vives-Cases is Senior Lecturer at the Department of Community Nursing, Public Health, Preventive Medicine and History of Science of the University of Alicante, Spain. Her research tasks have been focused mostly on social determinants of violence against women in general and among immigrant and ethnic minority women, in the Spanish context in particular. Ann Öhman has a PhD in Public Health and is currently Professor of Gender Studies at Umeå Centre for Gender Studies, Umeå University, Sweden. She is also affiliated to the Department of Clinical Medicine and Public Health; Epidemiology and Global Health, Umeå University. Her research deals with gender and health, gender-based violence, and gendered work in health care institutions.

How to submit: You can find the instructions for authors at: (www.globalhealthaction.net/index.php/gha/about/submissions#authorGuidelines).

Please submit your work using the online submission system, and under the Journal section, select ‘Special issues: Gender and health inequalities: intersections with other relevant axes of oppression’. This call will be open until the end of August 2016, and manuscripts will be published as soon as they are accepted.

*Carmen Vives-Cases* Department of Community Nursing, Preventive Medicine and Public Health and History of Science Alicante University, Alicante, Spain CIBER of Epidemiology and Public Health Barcelona, Spain *Malin Eriksson* Epidemiology and Global Health Unit Department of Public Health and Clinical Medicine Faculty of Medicine, Umeå University Umeå, Sweden *Isabel Goicolea* Epidemiology and Global Health Unit Department of Public Health and Clinical Medicine Faculty of Medicine, Umeå University Umeå, Sweden *Ann Öhman* Epidemiology and Global Health Unit Department of Public Health and Clinical Medicine Faculty of Medicine, Umeå University Umeå, Sweden Umeå Centre for Gender Studies Umeå University Umeå, Sweden

